# Large‐scale deep proteomic analysis in Alzheimer's disease brain regions across race and ethnicity

**DOI:** 10.1002/alz.14360

**Published:** 2024-11-13

**Authors:** Fatemeh Seifar, Edward J. Fox, Anantharaman Shantaraman, Yue Liu, Eric B. Dammer, Erica Modeste, Duc M. Duong, Luming Yin, Adam N. Trautwig, Qi Guo, Kaiming Xu, Lingyan Ping, Joseph S. Reddy, Mariet Allen, Zachary Quicksall, Laura Heath, Jo Scanlan, Erming Wang, Minghui Wang, Abby Vander Linden, William Poehlman, Xianfeng Chen, Saurabh Baheti, Charlotte Ho, Thuy Nguyen, Geovanna Yepez, Adriana O. Mitchell, Stephanie R. Oatman, Xue Wang, Minerva M. Carrasquillo, Alexi Runnels, Thomas Beach, Geidy E. Serrano, Dennis W. Dickson, Edward B. Lee, Todd E. Golde, Stefan Prokop, Lisa L. Barnes, Bin Zhang, Varham Haroutunian, Marla Gearing, James. J Lah, Philip De Jager, David A Bennett, Anna Greenwood, Nilüfer Ertekin‐Taner, Allan I. Levey, Aliza Wingo, Thomas Wingo, Nicholas T. Seyfried

**Affiliations:** ^1^ Emory University School of Medicine Atlanta Georgia USA; ^2^ Department of Neuroscience Mayo Clinic Florida Jacksonville Florida USA; ^3^ Sage Bionetworks Seattle Washington USA; ^4^ Department of Genetics and Genomic Sciences Icahn School of Medicine at Mount Sinai New York New York USA; ^5^ Mount Sinai Center for Transformative Disease Modeling Icahn School of Medicine at Mount Sinai New York New York USA; ^6^ New York Genome Center New York New York USA; ^7^ Banner Sun Health Research Institute Sun City Arizona USA; ^8^ Center for Neurodegenerative Disease Research University of Pennsylvania Philadelphia Pennsylvania USA; ^9^ University of Florida Gainesville, 100 Academic Advising Center Gainesville Florida USA; ^10^ Rush Alzheimer's Disease Center Rush University Medical Center Chicago Illinois USA; ^11^ Columbia University Irving Medical Center New York New York USA; ^12^ Department of Neurology Mayo Clinic Florida Jacksonville Florida USA

**Keywords:** Alzheimer's disease, data descriptor, diversity, precision medicine, proteome, proteomics

## Abstract

**INTRODUCTION:**

Alzheimer's disease (AD) is the most prevalent neurodegenerative disease, yet our comprehension predominantly relies on studies within non‐Hispanic White (NHW) populations. Here we provide an extensive survey of the proteomic landscape of AD across diverse racial/ethnic groups.

**METHODS:**

Two cortical regions, from multiple centers, were harmonized by uniform neuropathological diagnosis. Among 998 unique donors, 273 donors self‐identified as African American, 229 as Latino American, and 434 as NHW.

**RESULTS:**

While amyloid precursor protein and the microtubule‐associated protein tau demonstrated higher abundance in AD brains, no significant race‐related differences were observed. Further proteome‐wide and focused analyses (specific amyloid beta [Aβ] species and the tau domains) supported the absence of racial differences in these AD pathologies within the brain proteome.

**DISCUSSION:**

Our findings indicate that the racial differences in AD risk and clinical presentation are not underpinned by dramatically divergent patterns in the brain proteome, suggesting that other determinants account for these clinical disparities.

**Highlights:**

- We present a large‐scale proteome (∼10,000 proteins) of DLPFC (998) and STG (244) across AD cases.
- About 50% of samples were from racially and ethnically diverse brain donors.
- Key AD proteins (amyloid and tau) correlated with CERAD and Braak stages.
- No significant race‐related differences in amyloid and tau protein levels were observed in AD brains.
- AD‐associated protein changes showed a strong correlation between the brain proteomes of African American and White individuals.
- This dataset advances understanding of ethnoracial‐specific AD pathways and potential therapies.

## 1 BACKGROUND

Alzheimer's disease (AD) presents a significant global health challenge, with its prevalence affecting millions worldwide.^1^, ^2^ Notably, African Americans (AAs) and Hispanic Americans (HAs) are almost twice as likely as non‐Hispanic Whites (NHWs) to develop AD and/or other dementias.^3^, ^4^ The mechanisms contributing to this disparity are multifaceted, including a combination of genetic differences, as well as societal and environmental inequities that disproportionately affect minoritized populations.^4^, ^5^, ^6^, ^7^, ^8^, ^9^, ^10^, ^11^, ^12^ Emerging evidence suggests differences in some molecular measures, such as lower cerebrospinal fluid (CSF) levels of tau and other synaptic proteins in African Americans with AD compared to NHWs.^13^, ^14^, ^15^ A recent CSF proteome analysis across diverse populations with AD highlighted potential racial differences in the molecular basis of AD.^14^ Specifically, that study found that proteins involved in cytoskeletal function and gluconeogenesis are less abundant in the CSF of African Americans with AD, suggesting that variations in biomarkers, including tau and amyloid beta (Aβ), might reflect broader, race‐specific differences in the brain proteome. These findings underscore the importance of further research into the proteomic changes in AD, as they could provide critical insights into how the disease manifests differently across populations, potentially guiding more personalized approaches to diagnosis and treatment. There is, however, a significant gap in our understanding of the ethnoracial disparities inherent in the pathophysiology of AD. To address this gap in knowledge, the National Institute on Aging and Accelerating Medicines Partnership in AD (AMP‐AD) sought to promote inclusivity in multi‐omics AD research and to unravel unique molecular signatures and pathways.^16^

Proteins serve as optimal markers for understanding “proteinopathies” like AD and other neurodegenerative disease due to their proximity to pathologic and phenotypic changes in disease.^17^ With the advancement of multiplex isobaric tandem mass tags (TMTs), off‐line fractionation, and high‐resolution mass spectrometry (MS), proteomic datasets are now approaching the scale and depth of transcriptomic datasets.^18^, ^19^, ^20^, ^21^, ^22^, ^23^ However, a comprehensive and detailed proteome dataset of the human brain spanning various regions, races, and ethnicities is lacking. Such a resource could uncover race‐specific protein differences, shedding light on distinct pathophysiologies, biomarkers, and potential therapeutic targets.

Using TMT coupled with MS (TMT‐MS), we report here the deep proteome (∼10,000 proteins) of the *post mortem* dorsolateral prefrontal cortex (DLPFC) from 998 individuals and the superior temporal gyrus (STG) from 244 individuals across control and pathologically defined AD cases. Of these, approximately 50% of the samples were from racially and ethnically diverse donors. Implementing a methodology for quality control (QC) measures, we ensured the removal of batch‐related variations from the dataset using a previously published method.^24^, ^25^ Subsequently, variance partition analyses were carried out to identify top proteins based on individual characteristics, such as sex, race, and AD diagnosis, across both DLPFC and STG tissues. Through TMT‐MS, we characterized core proteins associated with AD pathology, including amyloid precursor protein (APP) and the microtubule‐associated protein tau (MAPT), revealing a clear correlation of APP and MAPT levels with Consortium to Establish a Registry for Alzheimer's Disease (CERAD) and Braak stages. Furthermore, we show consistency between Apolipoprotein 4 (apoE4) protein levels with *APOE4* genotyping in the brain.

Furthermore, we present an analysis exploring regional differences in brain proteome, focusing on tau tangles and amyloid plaques. Our findings demonstrated a consistent association between proteins correlated with APP and MAPT across both the DLPFC and STG regions. Additionally, we conducted a global differential abundance analysis of the proteome, including AD hallmark protein fragments such as Aβ42, Aβ40, and specific tau domains, with a focus on racial differences. This analysis revealed a shared pattern of global differences in the AD proteome, with only a few distinct protein abundance variations in African Americans. In addition, no differential abundance was observed in Aβ42, Aβ40, MAPT, or most MAPT domains. This comprehensive large‐scale proteomic dataset establishes the foundation for a better understanding of ethnoracial‐specific protein modulations, distinct pathways, pathologies, biomarkers, and potential therapeutic targets in AD.

## 2 METHODS

### 2.1 Brain tissue collection

The proteomics data utilized in this study were a part of the AMP‐AD Diversity Initiative, a collaborative effort involving multiple research sites. The comprehensive dataset includes information from different multi‐omics data, including proteomics, genomics, and metabolomics. While the data generation and case selection analysis has been extensively described in the data descriptor manuscript,^26^ this study specifically focuses on database search, QC, and technical validation of the proteomics data.

1 **RESEARCH IN CONTEXT**

1. **Systematic review**: Large‐scale unbiased quantitative proteomics analysis of AD brain in a racially and ethnically diverse population is lacking.
2. **Interpretation**: We provide the largest ethnoracially diverse proteomic dataset to date, focusing on two distinct regions in the brain. AD‐related elevations in tau and amyloid levels are consistent across self‐identified racial groups. A global analysis of AD‐associated protein changes showed a strong correlation between the brain proteomes of African American and White individuals, with few distinct differences in each racial group.
3. **Future directions**: Combining the comprehensive proteomic dataset presented in this study with paired transcriptomics and genomics data in the future, holds the promise to unveil the intricate network of molecular targets and biomarkers that contribute to the multifaceted nature of AD pathogenesis across diverse populations.

In brief, brain samples were collected with the involvement of four institutions or data contribution sites: Mayo Clinic, Rush University, Mount Sinai University Hospital and Emory University. The goal of this initiative is to include diverse contributions from African American and Latino American populations. Each of the data contribution sites gathered brain samples from affiliated brain banks, cohort studies, and AD Research Centers (ADRCs) and were sent to Emory proteomics core for proteomic processing. A total of 1105 DLPFC tissues from 998 individuals were sent from all four data contribution sites including *n *= 129 from Emory University (including 22 samples from University of Pennsylvania), *n* = 399 from Mayo Clinic, *n* = 205 from Mount Sinai University Hospital, and *n* = 372 from Rush University. Frontal brain tissues from each contribution site were processed separately from the others. In addition, among Emory samples, 26 from Mount Sinai University were replicated, and among Mayo Clinic samples, 81 were replicated from Emory University samples.

A total of 280 STG tissues from 244 individuals were obtained from Emory University (*n* = 129) and Mayo Clinic (*n* = 151), and both were processed simultaneously.

### 2.2 Tissue homogenization, protein digestion, TMT peptide labeling, and liquid chromatography with tandem mass spectrometry

Tissue homogenization, protein digestion, TMT peptide labeling, pH fractionation, and liquid chromatography with mass spectrometry (LC‐MS/MS) are described in detail elsewhere.^26^ In brief, all samples were homogenized using 8 M urea lysis buffer mixed with HALT protease and phosphatase inhibitor in a Bullet Blender (NextAdvance). Samples were then sonicated and centrifuged, and the supernatants were collected for further analysis. A bicinchoninic acid (BCA) assay (Pierce) was used for protein concentration measurements. For protein digestion, initially, equal amounts of protein from each sample were aliquoted and pooled to create a global pooled internal standard (GIS) for each TMT batch. Therefore, the GIS in each batch made by combining portions from all samples within that batch. Then 100 µg of each sample was aliquoted and normalized across samples. Proteins were reduced with the addition of 1 mM dithiothreitol (DTT) followed by 5 mM iodoacetamide (IAA) alkylation. Samples were mixed with lysyl endopeptidase (Wako) at 1:100 (w/w) for overnight digestion. Further digestion was carried out by the addition of 50 mM ammonium bicarbonate at seven‐fold dilution, and trypsin (Promega) was added at 1:50 (w/w) for 16 h. Next, samples were acidified by adding (vol/vol) formic acid (FA) to the concentration of 1% and (vol/vol) trifluoroacetic acid (TFA) to a concentration of 0.1%. Protein solutions were then desalted with a 30 mg Hydropilic‐Lipophilic Balanced column (Oasis), rinsed and washed with 1 mL 50% (vol/vol) acetonitrile (ACN), and equilibrated with 2×1 mL 0.1% (vol/vol) TFA. Two volumes of 0.5 mL 50% (vol/vol) CAN were used for sample elution followed by dehydration using SpeedVac. For TMT labeling, each brain peptide digest was resuspended in 75 µL of 100 mM triethylammonium bicarbonate (TEAB) buffer, and 5 mg of TMT reagents was dissolved in 200 µL of acetonitrile (ACN). Then 100 µg of peptide samples were aliquoted and resuspended in 100 µL TEAB buffer. After bringing the TMT reagents to room temperature and mixing with ACN, 41 µL of the TMT solution was added to each peptide solution and incubated for 1 h. The reaction was stopped with the addition of 8 µL of 5% hydroxylamine. The labeled samples were then combined, concentrated using a SpeedVac, and diluted with 0.1% TFA. After acidification, the peptides were desalted using a C18 Sep‐Pak column, washed, and eluted with 50% ACN. The eluates were dried using a SpeedVac. Dried peptides were resuspended in a high‐pH loading buffer (0.07% NH4OH, 0.045% FA, 2% ACN) and loaded onto a Waters Bridged Ethylene Hybrid column. HPLC systems Thermo Vanquish and Agilent 1100 were used for fractionation at a flow rate of 0.6 mL/min with a 25‐min gradient. The mobile phases were 0.0175% NH4OH, 0.01125% FA, and 2% ACN for solvent A and 0.01125% FA, 0.0175% NH4OH, and 90% ACN for solvent B. In total, 192 fractions were collected, pooled into 96 or 48 fractions, depending on cohort, and then dried with a SpeedVac. The same methods were used for fractionating additional cohorts offline.

TMT‐MS was carried out by resuspending the dried fractions in loading buffer (0.1% FA, 0.03% TFA, 1% ACN) and analyzing them using LC‐MS/MS. Peptide separation was achieved on a self‐packed C18 column (1.9 µm, 25 cm × 75 µm ID) using a Dionex UltiMate 3000 RSLCnano system with a 180‐min gradient at a flow rate of 225 nL/min. After performing full scans (m/z 350 to 1500, 120,000 resolution) using the mass spectrometer's data‐dependent mode, the MS/MS scans were performed using higher energy collision‐induced dissociation (HCD).

### 2.3 Database searches and protein quantification

All raw files underwent a database search using Fragpipe (version 19.0) for DLPFC and STG datasets, separately. The database search parameters have been described elsewhere.^27^, ^28^ Initially, mzML files were created from the original MS  .raw files for frontal (6479 raw files across 72 batches) and temporal regions (1824 raw files across 19 batches) using the ProteoWizard MSConvert tool (version 3.0) with specific options, including “Write index,” “TPP compatibility,” “Use zlib compression,” and a “peakPicking” filter setting.

Following the creation of mzML files for each set, they were subjected to a search using MSFragger (version 3.5). The human proteome database used contained 20,402 sequences (Swiss‐Prot, downloaded February 11, 2019) along with corresponding decoys and common contaminants. The sequences included additional specific peptide sequences for the APOE ε4 and APOE ε2 alleles.^29^

The search settings included a precursor mass tolerance of −20 to 20 ppm, a fragment mass tolerance of 20 ppm, mass calibration, parameter optimization, isotope error set to −1/0/1/2/3, strict‐trypsin enzyme specificity, and allowance for up to two missed cleavages. Fully enzymatic cleavage type, peptide length (7 to 50), and peptide mass (200 to 5000 Da) criteria were defined. Variable modifications included oxidation on methionine, N‐terminal acetylation on protein, and TMTpro modification on the peptide N‐terminus, with a maximum of three variable modifications per peptide. Static modifications comprised isobaric TMTpro (TMT16) modifications on lysine, along with carbamidomethylation of cysteine.

In the Post‐MSFragger (version 3.6) search, Percolator^30^ was used for Peptide‐Spectrum Match validation, succeeded by Philosopher (version 4.6.0) for protein inference using ProteinProphet and false discovery rate (FDR) filtering. Reports containing quantified peptides and UniprotID‐identified proteins with FDR < 1% were generated.

Following the initial protein search and QC steps for protein search, we conducted a targeted re‐search of our proteomics data, focusing on peptides of interest, including four main domains of MAPT and the amyloid cleavage fragments Aβ40 and Aβ42, as described elsewhere.^31^ The domain‐level search included independent sequences for MAPT (P10636_8) targeting the N‐terminal (amino acids, 1 to 126), proline‐rich domain (PRD) (amino acids 127 to 242), microtubule binding region (MTBR) (amino acids, 243 to 369), and C‐terminal region (amino acids 370 to 441).

### 2.4 Data analysis and QC

In this study, the analysis was performed using the traits provided by SAGE at https://www.synapse.org/Synapse:syn51757646. These data are available for general research use following the access guidelines provided at the following link: https://adknowledgeportal.synapse.org/Explore/Studies/DetailsPage/StudyData?Study=syn51732482.

The data analysis, using R statistical software (version 4.3.2), followed a three‐step process:

#### 2.4.1 Step 1: Preprocessing for missing values

Proteins with missing data in less than 50% of the samples were retained as described.^21^, ^32^ The ratio of protein abundance to the total protein abundance for each sample was calculated to adjust for sample loading differences. Subsequently, a log_2_ transformation was applied to enhance the normality of the distribution of protein abundance, addressing potential skewness and stabilizing variance across samples.

#### 2.4.2 Step 2: Outlier detection and removal

Iterative principal component analysis (PCA) was employed to identify and eliminate samples more than four standard deviations from the mean of either the first or second principal component, as previously described.^33^, ^34^ Multiple iterations of PCA were conducted, with outliers from each round being systematically removed before initiating the subsequent iteration.

#### 2.4.3 Step 3: Accounting for batch effect

A linear regression model was fitted to estimate the effect of protein sequencing batch. We then regressed out the batch effect from the protein abundance before the next step of analysis to minimize batch effects and enhance the reliability of downstream analyses.

This process was explained in previous studies.^24^, ^25^

### 2.5 Variance partition analysis

To explore how different traits influence protein abundance, we applied variance partition analysis (VPA).^35^ This model allowed us to break down the overall variability in our data and determine how much each trait, such as sex, race (comparing African American or Black individuals to all other races), and AD diagnosis (comparing AD to all other diagnoses), contributes to the observed differences. We also accounted for other residual factors in our models. The model we used can be represented by the following equation:

yij=μ+αi+βj+γk+∈ijk.

In this equation, yij represents the abundance of a specific protein, μ is the overall mean protein abundance, αi denotes the effect of sex, βj represents the effect of being African American or Black, γk accounts for the effect of AD diagnosis, and ϵijk is the residual error that includes other unexplained sources of variation.

We estimated the contribution of each factor to the total variance using a linear mixed‐effects model, which allowed us to break down the observed variability into variance components for each factor. These contributions were then quantified as proportions of the total variance.

### 2.6 Data QC for APP and MAPT domains and age and sex regression

We applied two‐way median polish with TAMPOR to correct for batch effects, as described elsewhere.^14^, ^31^, ^36^ To ensure that amyloid fragments and MAPT domains were not affected by any potential confounding effects other than race and AD diagnosis, the data were bootstrapped to adjust for age and sex in follow‐up differential analysis.

### 2.7 Differential abundance analysis

A one‐way ANOVA followed by Tukey's post hoc test for multiple comparisons was conducted on two sets (Control‐White vs AD‐White and Control‐African American vs AD‐African American) to identify proteins with differential abundance across diagnoses within each racial group. The findings were then visualized as volcano plots using the ggplot2 package in R, as previously described.^14^, ^31^

### 2.8 Shiny APP

An interactive web‐based Shiny APP (https://telomere.biochem.emory.edu/diversity/) is provided to facilitate analysis of this dataset. A Shiny app is an interactive web application built in R that allows users to explore and visualize large data. In our study, one of the features of the Shiny app is a volcano plot comparing protein abundance between AD and control samples, with options to interactively select proteins and generate corresponding boxplots. Users can explore data by traits like race, APOE genotype, and sex, with race‐specific comparisons. The app also includes a bookmarking feature to save and share specific views, making it a powerful tool for understanding proteomic changes in AD.

## 3 RESULTS

### 3.1 Cohort characteristics

**FIGURE 1:**
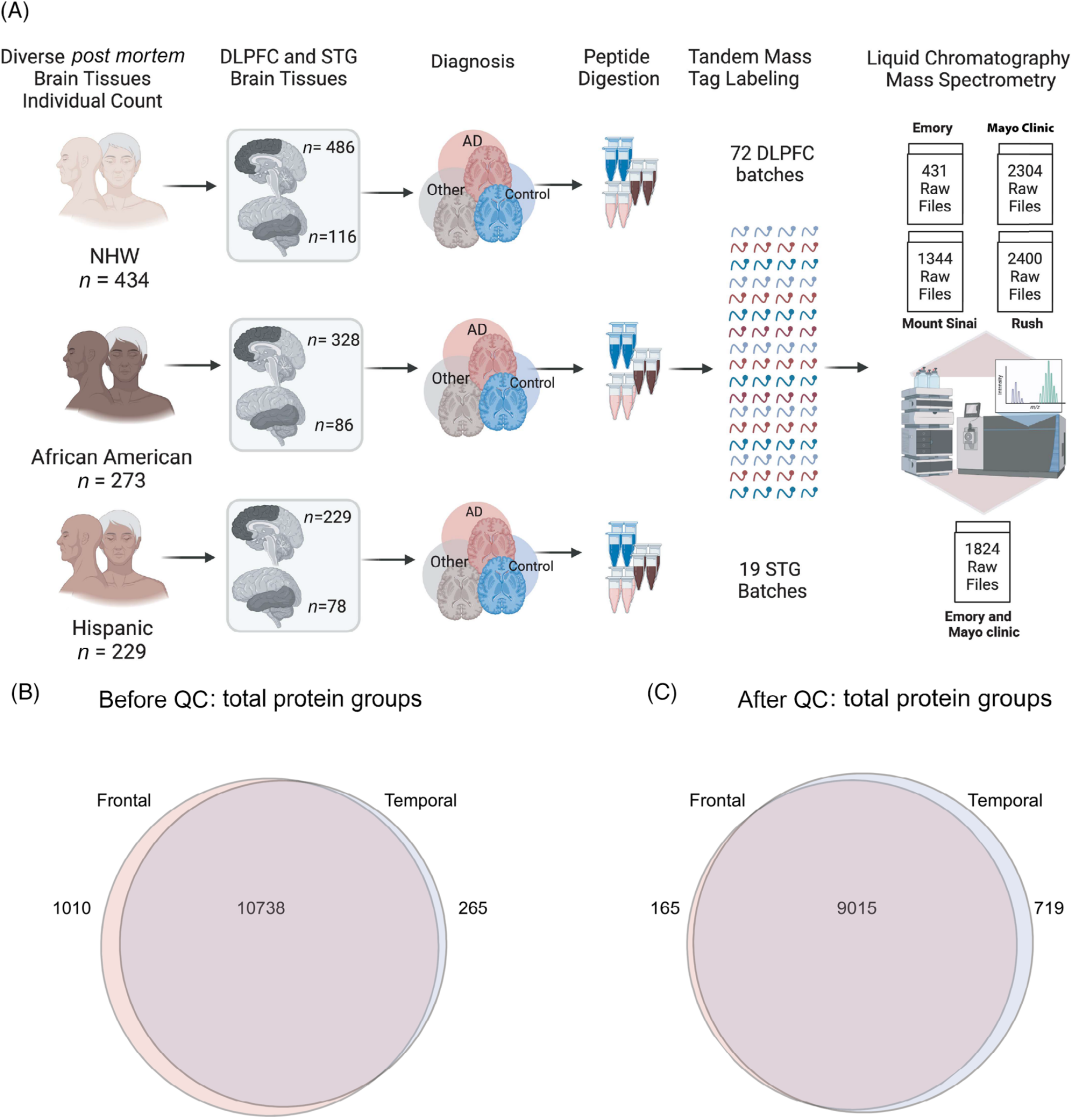
(A) Schematic illustrating cohort characteristics and experimental workflow for TMT‐MS of human brain proteome across frontal and temporal brain tissue samples. This study incorporated a total of 1105 DLPFC brain tissues from 998 individuals, categorized as follows: 486 NHW, 328 African American, 229 Latino American, and others as applicable. These samples were sourced from four prominent data distribution sites: Emory University, Mayo Clinic, Rush University, and Mount Sinai University Hospital. Additionally, 280 STG tissues from a subset of 244 individuals were included, with 116 NHW, 86 African American, 78 Hispanic, and others as applicable. STG samples were obtained from a racially diverse set of specimens originating from Mayo Clinic and Emory, distributed across 19 batches. Tissues underwent an experimental pipeline involving protein digestion, batch randomization, TMT labeling, fractionation, and subsequent MS measurements. A total of 72 DLPFC batches were processed, comprising nine batches from Emory, 24 from Mayo Clinic, 14 from Mount Sinai, and 25 from Rush (comprising a total of 72 batches). Batches were randomized to ensure a representative and diverse dataset. The output included a total of 6479 raw files for DLPFC samples and 1824 raw files for STG. (B) Venn diagram of total number of proteins quantified from DLPFC and STG samples. A total of 11,748 protein groups were identified from DLPFC and 11,003 from STG samples, with 10,738 shared protein groups. (C) Venn diagram of total protein from DLPFC and STG samples after QC across all samples. 9180 protein groups were identified from DLPFC samples and 9734 from STG, with 9015 shared protein groups. DLPFC, dorsolateral prefrontal cortex; NHW, non‐Hispanic White; QC, quality control; STG, superior temporal gyrus; TMT‐MS, tandem mass tag mass spectrometry.

We analyzed a diverse set of brain samples (Figure 1A), which included 486 samples from 434 unique non‐Hispanic White (NHW) individuals, 328 samples from 273 unique non‐Hispanic Black or African American individuals, and 229 samples from 229 Hispanic/Latino non‐White, non‐Black (non‐African American) individuals. Additionally, the study included smaller groups from mixed or other racial backgrounds: 11 samples from Asian individuals, five samples from American Indian or Alaska Native individuals, and four samples from unknown racial or ethnic groups. In this study, our analysis primarily focused on racial differences, comparing White individuals directly with African Americans (irrespective of ethnicity). Out of 1105 DLPFC tissues, 645 were from AD brains, 250 were from controls, and 210 had other diagnoses or missing or unknown diagnoses.

The 280 STG samples included 116 samples from 116 unique NHW individuals, 86 samples from 77 African American individuals, and 78 samples from 51 Hispanic/Latino individuals. Out of 280 STG tissues, 178 samples were collected from AD brains, 87 from controls, 14 from other diagnosis, and one from someone with a missing or unknown diagnosis.

Samples were collected from different sites and underwent TMT‐MS in different batches. Collectively, TMT‐MS led to a total of 6479 raw files from DLPFC and 1824 raw files from STG (Figure 1A), with the distribution as follows: Emory University Frontal Cortex Cohort: 431; Mayo Clinic Frontal Cortex Cohort: 2304; Mount Sinai Frontal Cortex Cohort: 1344; Rush University Frontal Cortex Cohort: 2400; and Emory University and Mayo Clinic Temporal Cortex Cohort: 1824. The database search led to the identification of a total of 11,748 protein groups from DLPFC samples and 11,003 from STG samples, revealing a shared set of 10,738 protein groups (Figure 1B). Following the QC process, a total of 9180 proteins remained for the DLPFC and 9734 proteins for the STG, with an overlap of 9015 protein groups shared between the two brain regions (Figure 1C).

### 3.2 Proteomics data QC in frontal and temporal cortices

**FIGURE 2:**
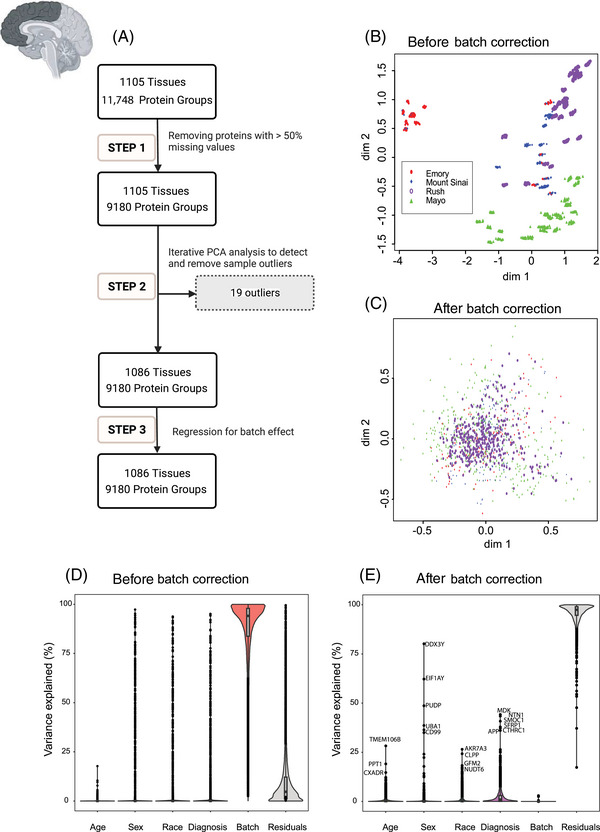
QC and batch correction for DLPFC tissue proteins. (A) QC workflow in three main steps. Step 1: Preprocessing for missing values: Only proteins with missing data in less than 50% of the samples were retained. The ratio of protein abundance to the total protein abundance for each sample was calculated to adjust for sample loading differences resulting in 9180 proteins being retained across 1105 samples. Subsequently, the data were log_2_ transformed. Step 2: Outlier detection and removal: Iterative PCA was employed to identify and eliminate sample outliers. After multiple rounds of PCA analysis, 19 outliers were identified and removed, leaving 9180 proteins across 1086 samples. Step 3: Batch effect regression: Variance attributable to batching was mitigated through regression of the 9180 proteins in 1086 samples. (B and C) MDS plot showing variation among samples (B) before correcting for batch and (C) after regressing for batch effect. The plot dimensions (dim 1 and 2) reveal distinctive clusters formed by samples by site – Emory (red), Mount Sinai (blue), Rush (purple), and Mayo (green) – with some scattering observed among samples before regressing for batch effect (B). The plot in (C) illustrates the successful removal of variance due to batch. After correcting for batch effects, samples from all four sites – Emory (red), Mount Sinai (blue), Rush (purple), and Mayo (green) – cluster together, indicating a more cohesive grouping (ie, the change in scale from B to C). The correction mitigates the dispersion observed in (B), highlighting the effectiveness of the batch correction procedure in harmonizing the sample distribution across different data distribution sites. (D and E) Variance partition analysis using experimental factors to evaluate the percentage of explained variance in proteomic samples. Violin plots before (D) and after (E) batch correction illustrate the distribution of explained variances in overall proteomic values. The *y*‐axis represents the percentage of explained variance, while the *x*‐axis depicts factors contributing to variance, such as age, sex, race, diagnosis, residuals, and batch. Notably, batch variance is present before batch correction, influencing the overall proteomic profile. Panel (E) displays the same factors on the *x*‐axis after batch correction. Significantly, the violin plot demonstrates a substantial reduction in variance associated with batch, ultimately reaching near zero percent after batch regression. Moreover, even after batch correction, factors such as age, sex, race, AD diagnosis, and other individual traits (residual) had levels of impact on protein abundance patterns. Each point on the violin plot represents a specific protein, with the corresponding name next to it. This underscores the efficacy of the correction procedure in eliminating batch‐related variability from the proteomic data. DLPFC, dorsolateral prefrontal cortex; MDS, multidimensional scaling; NHW, non‐Hispanic White; PCA, principal component analysis.

The analysis workflow for data QC is illustrated in the flowcharts of Figure 2A and Figure S1A in three main steps. For the DLPFC 19 outliers and for the STG two outliers were removed, leading to a total of 1086 samples in DLPFC and 278 samples in STG. The QC process also included steps to adjust for batch as described in *Methods*.

In large‐scale TMT‐MS proteomics studies, batch effects are inevitable due to technical reasons; this is especially problematic when processing large cohorts in multiple separate batches.^36^, ^37^

To investigate the variability associated with batch effects among DLPFC samples prior to normalization and batch correction, we employed multidimensional scaling (MDS). MDS is similar to PCA, which is used for visualizing high‐dimensional data in lower‐dimensional spaces.^38^ Before batch regression, distinctive clusters of samples from different sites were observed (Figure 2B). After batch correction, samples clustered together, indicating that the batch regression successfully gave an even distribution of data without regard to data distribution sites (Figure 2C). The effectiveness of batch correction was also assessed through variance partition analysis (VPA),^35^ which revealed that the percentage of variance in protein abundance explained by the batch was reduced to near zero after batch regression from >90% before correction (Figure 2D, E).

Similarly, to investigate the impact of batch on temporal cortex samples, MDS plots were utilized. The plots illustrated a distinct clustering by batch before QC, followed by an even distribution after QC (Figure S1B, S1C). In addition, batch variance revealed a high contribution to the proteomic profile before correction in variance partitioning (Figure S1D) and a substantial reduction in variance associated with batch after QC (Figure S1E).

### 3.3 Variance of protein abundance in frontal and temporal cortex explained by individual traits

**FIGURE 3:**
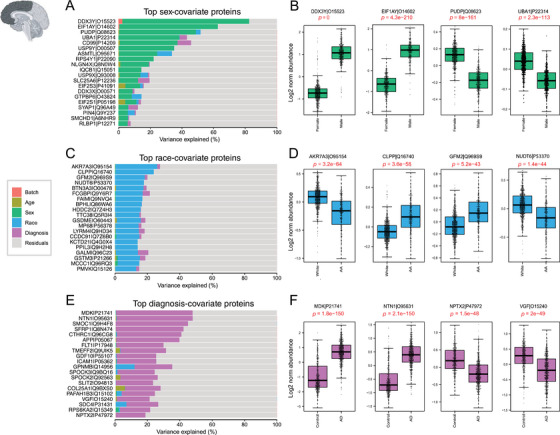
Variance explained by individual traits in DLPFC tissues. Bar plots (A, C, E) depict amount of variance explained by sex, race, and AD diagnosis across all DLPFC samples. (A) Top‐ranking proteins associated with sex in dataset were identified through variance partitioning and depicted as bar plots. Boxplots in (B) illustrate log2 normal abundance levels of four selected proteins exhibiting significant differences between males and females. These proteins serve as key indicators of sex‐related variations and are depicted with statistical significance (*p* < 0.05). (C) Bar plots of top‐ranking proteins associated with race differences in DLPFC dataset. Boxplots in (D) illustrate log2 normal abundance levels of four selected proteins demonstrating significant differences between African American individuals and other races (*p* < 0.05). (E) Bar plots identified top‐ranking proteins contributing to differences in diagnosis of AD within dataset. Boxplots in (F) display the log2 normal abundance levels of four selected proteins exhibiting significant differences between AD patients and controls, as well as other diagnostic categories (*p* < 0.05). AD, Alzheimer's disease; DLPFC, dorsolateral prefrontal cortex.

Variance partition analysis of DLPFC showed that a small proportion of the variance in protein abundance could be explained individually by sex, race, and diagnosis (Figure 3). Proteins such as CD99, PUDP, and UBA1, which are associated with the X chromosome and known to be highly abundant in females,^39^, ^40^ contributed significantly to the observed variance attributable to the sex of the donor (Figure 3A, Figure S2A). Similarly, EIF1Y, DDX3Y, and USP9Y, linked to the Y chromosome and known for their high levels in males,^39^, ^40^ also played a role in explaining the observed variance. Subsequent analysis confirmed significant differences (*p* < 0.05) in protein levels between males and females (Figure 3B, Figure S2B), further reinforcing the importance of sex as a determinant of proteomic variability in our dataset.

Key proteins associated with self‐reported African American race, such as BPHL, FAIM, GFM2, and CLPP, were identified through variance partition analysis in both frontal and temporal cortex samples (Figures 3E and S2C). Notably, proteins associated with African American race in the temporal cortex displayed a different rank order compared to frontal cortex proteins (Figure S2C). Further analysis highlighted significantly higher levels of proteins like GFM2 and CLPP in African American individuals. In contrast, the protein NUDT6 exhibited markedly lower levels within this demographic group (Figure 3D and Figure S2D). The variance explained by each of the variables for all the proteins in DLPFC is listed in Table S1.

A parallel analysis explored the extent to which AD diagnosis explained the variance in protein level within the frontal and temporal cortex. Consistent with the existing literature,^21^, ^41^, ^42^ top‐ranking proteins associated with AD, including APP, which has been shown to correlate with Aβ plaque burden in the brain,^21^ as well as other amyloid‐associated matrisome proteins, CTHRC1, SMOC1, MDK, and NTN1, exhibited significantly higher levels among AD cases^43^, ^44^ (**Figure 3E and** S2E). Notably, the percentage of variance contributed by individual proteins varied by region, for example, NDP and MACROD1 (Figure S2E). The variance partition values for all the STG proteins are listed in Table S2. This comprehensive analysis not only supports the technical validation of our proteomics data but also provides insights into the molecular basis of sex, race, and AD‐associated variations in human brain proteomic data.

### 3.4 Association between *APOE4* genotype and apoE4 protein abundance in human brain proteome

**FIGURE 4:**
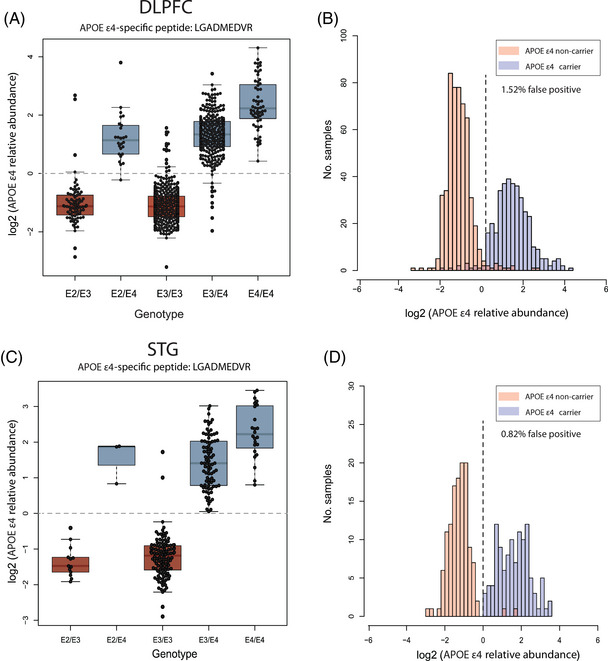
Association between APOE ε4 genotype and prototype across DLPFC and STG samples. (A) The boxplots of log2 normal abundance of APOE ε4 protein measured by TMT‐MS across each APOE genotype reveal a high APOE ε4 abundance among APOE ε4 carriers among 920 unique DLPFC tissue samples. (B) Histogram of APOE ε4 log2 normal abundance among DLPFC samples (*y*‐axis) across ε4 allele presence (red) and non‐presence (blue) (*x*‐axis). (C) The boxplots of log2 normal abundance of APOE ε4 protein measured by TMT‐MS across 244 STG unique tissue samples reveal a high APOE ε4 abundance among APOE ε4 carriers. (D) Histogram of APOE ε4 log2 normal abundance among STG samples (*y*‐axis) across ε4 allele presence (red) and non‐presence (blue) (*x*‐axis). High levels of APOE ε4 abundance were observed in cases with the ε4 allele combination in both cortices, with a few discrepancies between APOE ε4 genotyping and prototyping (purple) being depicted. These inconsistencies may be attributed to various factors, including mis‐genotyping or potential technical challenges in MS measurements, such as isotope impurity and low signal‐to‐noise ratio in specific samples. DLPFC, dorsolateral prefrontal cortex; STG, superior temporal gyrus; TMT‐MS, tandem mass tag mass spectrometry.

The *APOE* locus exists as three AD‐related variants (ε2, ε3, ε4), each associated with varying degrees of AD risk, with the *APOE* ε4 allele representing a major genetic risk factor for non‐dominantly inherited AD.^45^, ^46^ The apoE ε4 protein variant can be differentiated by a cysteine‐to‐arginine change that can be detected and measured at the peptide level.^47^ We therefore measured the abundance of an apoE4‐specific tryptic peptide (LGADMEDVR) and compared its detection to genotypic data for *APOE* across 920 unique individuals where genotyping information was available in DLPFC samples. A similar analysis was carried out on 244 unique donors of STG samples. As expected, given the unique change in protein sequence, the mean fold change of apoE4 protein abundance between *APOE ε4* carriers and non‐carriers was >8‐fold (Figure 4A and C). While it was expected that the apoE4 peptide signal would not be present in non‐*APOE* ε4 carriers, cases without an ε4 allele still exhibited detectable, lower signals for the peptide abundance. These signals could be attributed to errors in genotyping or background chemical noise, possibly stemming from TMT isotope impurity. Similarly, there was a limited number of false positive (non‐*APOE* ε4 carriers with discrete APOE4 signal) proteotypes (< 2.0%) observed between expected *APOE* genotype and apoE4 peptide levels, mainly in DLPFC samples. Specifically, we observed 14 individuals among the DLPFC samples and two individuals among the STG samples with homozygous *APOE* ε3 genotypes that had levels of apoE4 equivalent to individuals with *APOE* ε4 genotypes (Figure 4B and D). These samples could be removed from further analysis as appropriate. Nevertheless, approximately 98% of samples appeared to have the correct *APOE ε4* genotype based on apoE4 “proteotype.”

### 3.5 Correlation of amyloid and tau abundance in human brain proteome with AD neuropathology and other proteins

**FIGURE 5:**
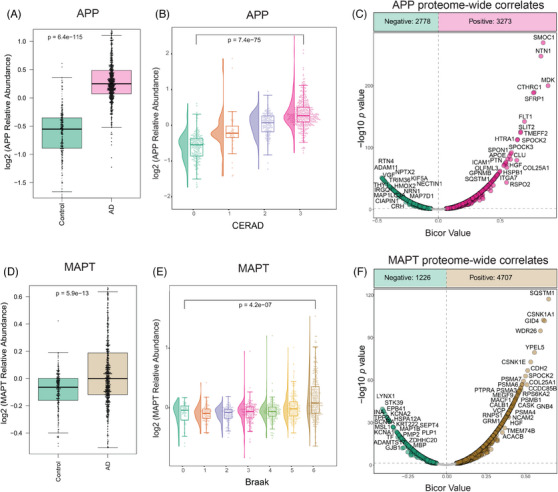
Correlation of proteomic measurements of tau (MAPT) and APP levels with Braak and CERAD scores, as well as with other proteins, in DLPFC. (A) Boxplots depicting relative abundance of APP across AD (pink) and control (green) in DLPFC tissue samples (adjusted ANOVA *p* value < 0.05). (B). Raincloud plots depict group differences in relative abundance of APP (*y*‐ axis) across distinct CERAD stages (*x*‐axis) in DLPFC tissues. The analysis revealed a stepwise increase in the median APP levels with ascending CERAD classifications, indicating a progressive trend in APP abundance corresponding to different CERAD groups (score 1: green, score 2: orange, score 3: purple, score 4: pink). (C) Bicor correlates to APP. The plot illustrates the results of a bicor pairwise correlation between APP and 9180 proteins in DLPFC region. Proteins with a significant positive correlation with APP (*p* < 0.05) are highlighted in pink, whereas those with a significant negative correlation (*p* < 0.05) are displayed in green. Proteins that did not show a significant correlation with APP are colored in gray. Among the 9180 proteins analyzed, 3273 showed a positive correlation with APP, and 2778 were negatively correlated with APP. (D) Boxplots depicting relative abundance of MAPT across AD (brown) and control (green) in DLPFC tissue samples (adjusted ANOVA *p* value < 0.05). (E) Raincloud plots illustrate group differences in relative abundance of MAPT (*y*‐axis) across distinct Braak stages (*x*‐axis) in DLPFC tissues. The Braak stages range from 0 to 6, with corresponding colors representing different stages (0: dark green, 1: orange, 2: purple, 3: pink, 4: light green, 5: yellow, 6: brown). Notably, the analysis highlights elevated MAPT levels at Braak stages 5 and 6, aligning with the expected increase in tau tangles in later stages of Braak in the frontal cortex. (F) Bicor pairwise correlation analysis between MAPT (tau) and 9180 proteins in the DLPFC region. Proteins with a significant positive correlation (*p* < 0.05) are shown in brown, while those with a significant negative correlation (*p* < 0.05) are shown in green. Of the proteins analyzed, 4707 had a positive correlation, and 1226 had a negative correlation with MAPT. Non‐significant correlations are depicted in gray. AD, Alzheimer's disease; APP, amyloid precursor protein; Bicor, biweight midcorrelation; DLPFC, dorsolateral prefrontal cortex; CERAD, Consortium to Establish a Registry for Alzheimer's Disease; MAPT, microtubule‐associated protein tau.

The pathological hallmarks of AD include the accumulation of Aβ plaques and hyperphosphorylated tau neurofibrillary tangles.^48^, ^49^ To examine the extent to which these known disease‐associated changes are reflected in the proteome, we assessed the levels of the APPand MAPT using TMT‐MS. APP has different isoforms, while in the brain it is typically expressed as a full‐length transmembrane protein consisting of 695 amino acid residues.^50^, ^51^ In proteomic studies of AD brains, the levels of APP have been shown to correlate well with Aβ plaque burden^52^, ^53^ driven in part by the Aβ region of the protein (described below). As expected, therefore, the proteomic quantification of APP revealed significantly higher levels in AD cases (*p* = 6.4e‐115) (Figure 5A). Furthermore, there is a stepwise increase in proteomic quantification of APP with increasing CERAD score, a measure of the extent of neuritic and diffuse plaques in brain tissue, (Figure 5B), demonstrating the ability of proteomic APP levels to quantitatively capture the biology underlying known diagnostic and pathologic measures of Aβ plaque burden.

To extend our understanding of the proteomic changes associated with the Aβ burden in the frontal cortex, we conducted biweight midcorrelation (bicor) analysis across all 9180 pairwise protein comparisons with APP for 980 unique donors (Figure 5C, Table S3). Consistent with previous findings, proteins associated with the matrisome (ie, module 42 of the brain consensus network^21^) exhibited the highest positive correlations with APP. MDK had the highest correlation (bicor = 0.88, *p* = 0), followed by SMOC1, NTN1, CTHRC1, and SFRP1, all known to colocalize with amyloid plaques and be enriched in amyloid plaque proteomes.^54^, ^55^, ^56^ In contrast, the strongest negative correlations with APP were observed for RTN4, ADAM11, VGF, NPTX2, and TRIM36. RTN4 had the highest negative correlation with APP. It is a reticulon protein, which plays a role in blocking BACE1 access to APP, thereby reducing Aβ production and potentially limiting amyloid plaque formation.^57^, ^58^ Similarly, NPTX2 and VGF, both associated with synaptic function and neuroprotection, have been identified as potential biomarkers with protective effects against AD.^59^, ^60^, ^61^ Additionally, TRIM36 is involved in the clearance of misfolded protein aggregates, including APP, consistent with its proposed role in mitigating amyloid burden in the brain.^62^ The full list of DLPFC proteins and the correlation values are available in Table S3.

Similarly, our proteomic analysis of MAPT that produces tau protein demonstrated, as expected, significantly elevated levels in AD cases (*p* = 5.9e‐13) (Figure 5D).^63^, ^64^ We assessed the association between the levels of measured MAPT in the frontal cortex and Braak staging.^65^ Proteomic measures of MAPT levels exhibited higher levels mainly in advanced Braak stages (stages 5 and 6) in cases where Braak staging was available (Figure 5E). Of note, the association between MAPT levels and Braak staging may be influenced by regional differences in tau pathology and staging. Specifically, neurofibrillary tangles are predominantly encountered in the neocortex in higher Braak stages. Therefore, the observed elevation in MAPT levels in individuals with advanced Braak stages could be attributed to the assessment of tau levels in neocortical samples, where tau tangle pathology is pronounced.

Similar to APP, we explored the correlation between MAPT abundance in the DLPFC proteome (Figure 5F). In our pairwise analysis, SQSTM1 showed the highest positive correlation with MAPT. Sequestosome‐1 protein (SQSTM1) is known for its role in the autophagic regulation of tau and has shown high enrichment in the fibrillary tangle proteome.^66^, ^67^, ^68^ Additionally, other proteins with strong correlations with MAPT included members of the CSNK1 family, which are involved in tau phosphorylation and clearance,^69^ as well as proteins associated with ubiquitination (GID4)^70^ and proteasome activity (PSM3, PSM6, PSM7). Interestingly, several proteins that are highly enriched in the amyloid‐associated module, including COL25A1 and SPOCK2, also exhibited strong positive correlations with tau.^21^ The most prominent proteins that were negatively correlated with tau included LYNX1, STK39, INA, EPB41L3, KCNA2, HSPA12A, TPP2, and FIS1. A number of these proteins have been associated with neuroprotective effects and synaptic plasticity.^71^, ^72^, ^73^

A similar analysis exploring correlates of APP and MAPT within the temporal cortex are presented in Figure S3. APP and MAPT levels were also significantly higher in the STG (*p* = 8.3e‐41 and *p* = 1.3e‐16, respectively) of individuals with AD compared to controls (Figure S3A, S3D). Moreover, there was a graded increase in APP and MAPT levels with CERAD (Figure S3B) and Braak scores (Figure S3E), respectively. A similar pattern emerged in the proteome‐wide correlation analysis of the STG, with a positive association of the matrisome proteins with APP abundance (Figure S3C). SQSTM1 and multiple members of the CSNK1 and PSM families also all presented as significantly and positively correlated with tau in the STG (Figure S3F). The full list of STG proteins and correlation values can be found in Table S4.

### 3.6 TMT‐MS quantification of APP and MAPT revealed no racial differences in AD

**FIGURE 6:**
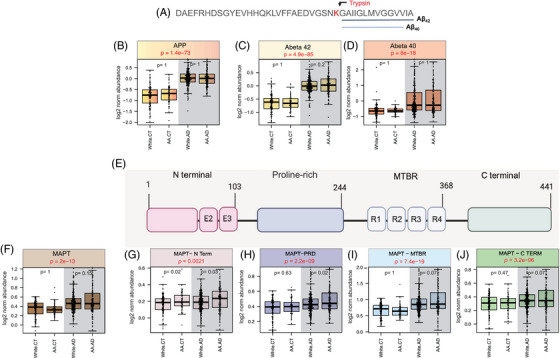
TMT‐MS quantification of APP, MAPT, and their specific fragments across White and African American cases with and without AD. (A) Schematic of APP fragments. Schematic representation of amino acid sequence of APP, with the specific C‐terminal tryptic cleavage sites leading to Aβ40 and Aβ42 measures via MS. The site of trypsin cleavage is marked by this red “K” (lysine) residue. (B‐D) Boxplots of log2 normalized abundance of APP (B), Aβ42 (C), and Aβ40 (D) in brain tissue samples from 980 unique individuals categorized by race and AD status. The four groups analyzed are White CT (*n* = 125), AA CT (*n* = 63), White AD (*n* = 281), and AA AD (*n* = 145). Data were adjusted for age and sex using the bootstrap method before analysis. The middle line in each boxplot shows the median, the box covers the range from the 25th to the 75th percentile, and the whiskers indicate the full range of the data. One‐way ANOVA was conducted to assess overall differences among the four groups, with significance set at *p* < 0.05. APP, Aβ42, and Aβ40 levels were significantly higher in AD cases compared to controls. Post hoc comparisons using Bonferroni correction did not reveal significant differences across race for these peptides. (E) Schematic of MAPT domains in 4R tau structure. Schematic representation of the four main domains of MAPT: N‐terminal, PRD, MTBR, and C‐terminal domain. (F‐J) MAPT domains. Boxplots of log2 normalized abundance of MAPT (F) and its domains: N‐terminal (G), PRD (H), MTBR (I), and C‐terminal (J) across the same four groups (White CT, AA CT, White AD, AA AD). Data were adjusted for age and sex using the bootstrap method before analysis. MAPT and all its domains showed significantly higher abundance in AD cases compared to controls. The PRD had a slightly higher abundance in AA with AD compared to White with AD (*p* = 0.02), and the N‐terminal domain showed a slight increase in AA individuals, both in controls (*p* = 0.02) and AD cases (*p* = 0.03). The MTBR was the main contributor to the MAPT signal in AD cases. The *p* values from these analyses are marked on the graphs. AA, African American; AD, Alzheimer's disease; APP, amyloid precursor protein; CT, control; MAPT, microtubule‐associated protein tau; MTBR, microtubule binding region; PRD, proline‐rich domain; TMT‐MS, tandem mass tag mass spectrometry.

Our analysis across both the DLPFC and STG regions demonstrated a consistent pattern where increased levels of proteomic measurements of APP and MAPT correlated with established AD pathologic and diagnostic scores. The proteome‐wide correlation analyses highlight the association of matrisome proteins with APP and proteins involved in autophagy and phosphorylation of tau, emphasizing their roles in the molecular mechanisms underlying AD. Emerging research has indicated that Aβ and tau peptides, identified as AD biomarkers, may differ across race.^13^, ^14^, ^15^, ^74^ Despite similar levels of cognitive decline, African Americans have been shown to have lower levels of certain CSF biomarkers compared to White individuals.^13^, ^74^ Using brain proteomes from diverse donors, we sought to determine whether the observed differences in the CSF reflect variation in the underlying pathology of AD in the brain. We first determined the proteomic abundance of MAPT and APP across race and AD diagnosis (Figure 6). We then quantified specific protein domains of APP and MAPT, stratifying our samples by race. To control for potential confounding effects of age and sex, we regressed the protein abundances for age and sex.

APP undergoes cleavage in two C‐terminal sites to form Aβ40 and Aβ42 (Figure 6A). The two amino acids at the C‐terminal end of Aβ42 – isoleucine (Ile) and alanine (Ala) – increase its tendency to aggregate and form Aβ plaques, making it more pathogenic compared to Aβ40.^75^ Aβ42 is a major component of neuritic plaques, whereas Aβ40 is often found in cerebral amyloid angiopathy (CAA), where it is typically deposited in blood vessel walls.^76^, ^77^ We determined the differential abundance of APP (Figure 6B), Aβ42 (Figure 6C), and Aβ40 (Figure 6D) across White and African American brain samples in AD or control groups. As expected, our analysis revealed a significantly higher abundance of APP and Aβ42 and Aβ40 peptides among AD brains compared to controls, however, without a significant race‐associated difference.

Tau protein is expressed and ultimately translated from the MAPT gene, and its accumulation is another pathologic hallmark of AD. When hyperphosphorylated, tau aggregates into neurofibrillary tangles, which strongly correlate with cognitive decline in AD.^78^, ^79^ Earlier studies showed that African Americans with AD had reduced levels of CSF tau and phosphorylated tau (p‐tau) compared to their White counterparts.^13^, ^14^, ^15^, ^74^ Our analysis revealed that total brain MAPT protein levels did not differ significantly between African Americans and White individuals (Figure 6F). We subsequently re‐analyzed our proteomic dataset to assess differences in MAPT‐specific protein domains, including the N‐terminal domain, PRD, MTBR, and C‐terminal domain (Figure 6E). The MTBR, located between amino acids 243 and 369, contains microtubule‐binding repeats and forms the core of the insoluble aggregates found in the AD brain, as observed in cryo‐EM studies.^80^, ^81^ Our analysis of these MAPT domains revealed significantly higher levels in AD brains, regardless of race, with the MTBR and C‐terminal domain showing the largest increase, consistent with previous observations^82^ (Figure 6G‐I). Interestingly, there was a slight but nominally significant increase in the N‐terminal domain and PRD in African Americans compared to Whites, observed in either AD cases or both control and AD cases. Nevertheless, while there were minor differences across those domains, the core proteomic signatures of tau (MAPT and MTBR domain) associated with tangle pathology (Braak) in the brain were similar in African Americans and White individuals.

### 3.7 Differential protein abundance across race in AD brain proteomes reveals a convergence of predominantly shared changes

**FIGURE 7:**
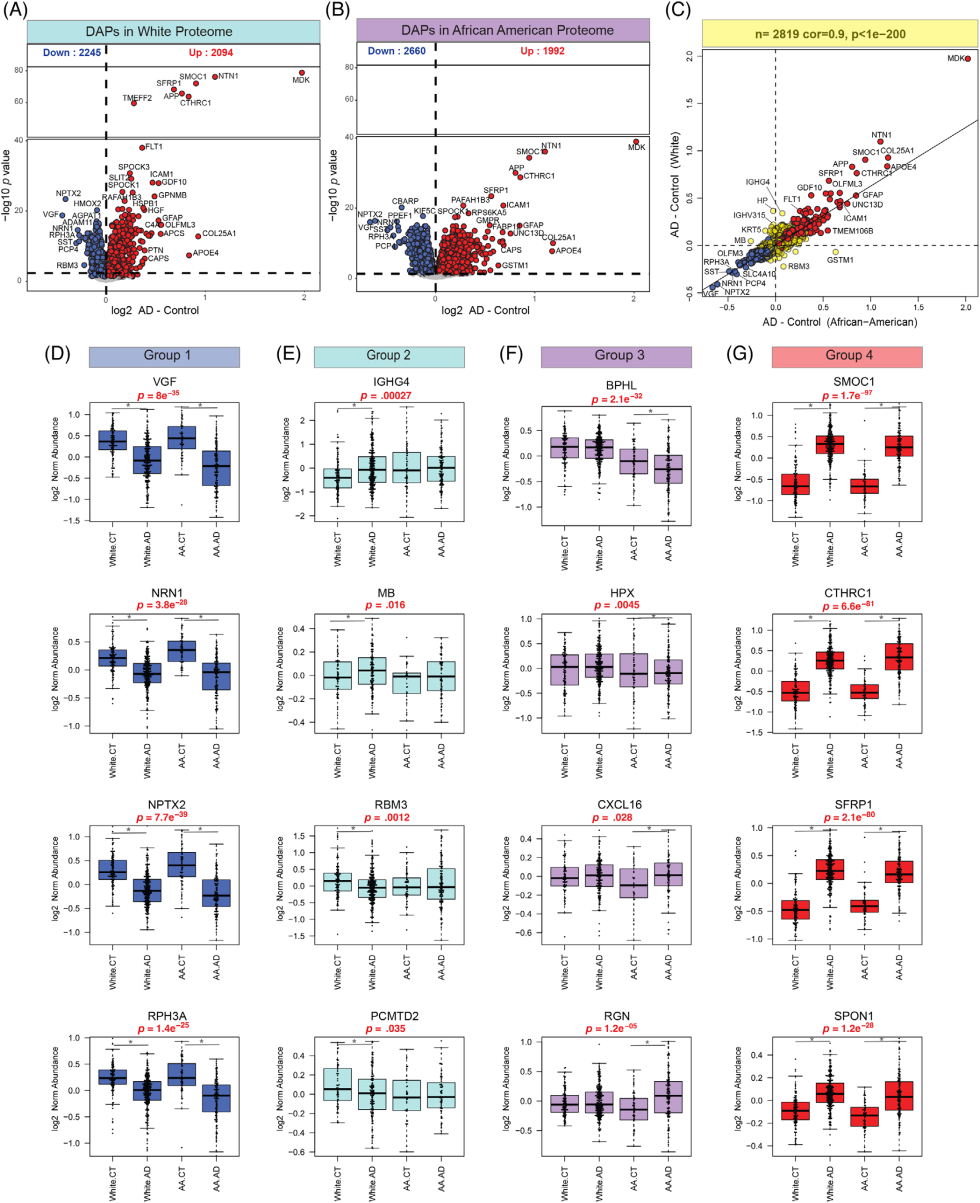
Global differential protein abundance between AD and control across racial groups. (A and B) Volcano plots displaying log2 FC (*x*‐axis) against one‐way ANOVA with Tukey correction‐derived −log10 *p* value (*y*‐axis) for all proteins (*n*  =  9180) comparing AD versus controls in White (A) and AA (B) proteomes. Proteins that are significantly more abundant in AD are presented in red, those significantly less abundant in blue, and proteins with non‐significant changes are presented in gray. (C) Scatter plot showing correlation between FC of all DAPs (*n* = 2819) found to be significant within the AA proteome (*x*‐axis) compared to the White proteome (*y*‐axis). The FCs were strongly correlated (bicor  =  0.9, *p*  < 1e‐200), regardless of whether the DAP was significant in one (yellow) or both proteomes (red: significantly higher abundance in AD, blue: significantly lower abundance in AD). (D‐G) Boxplots illustrating log2 FC relative abundance of representative proteins for four main categories across race and AD diagnosis: (D) DAPs in AD compared to control in both White and AA proteomes. (E) Proteins showing differential abundance across AD only in White individuals. (F) DAPs in only AA proteome across AD. (G) Proteins with a high abundance in AD compared with controls, independent of race. Boxes represent median and IQRs, and whiskers represent minimum and maximum data points within 1.5 times the IQR. *P* < 0.05 was considered significant. AA, African American; AD, Alzheimer's disease; Bicor, biweight midcorrelation; DAP, differentially abundant proteins; FC, fold change; IQR, interquartile range.

Global differential abundance analysis was performed to identify changes in the AD brain proteome with race (Figure 7A, B; Tables S5 and S6). Consistent with previous proteomic analyses of AD brain tissue, there was a significant increase in key proteins associated with AD pathology, such as matrisome‐associated proteins (eg, MDK, SMOC1, APOE, APP), as well as proteins involved in neuroinflammation (eg, GFAP, ICAM1) and synaptic function (eg, VGF, NPTX2) in both African Americans and White individuals with AD compared to controls in the same racial group. A scatter plot illustrates the correlation of differentially abundant proteins (DAPs) in AD from African Americans and White populations (Figure 7C), showing strong overall agreement in the direction and magnitude of change (bicor = 0.9, *p* < 1e‐200; Figure 7D,G).

In addition, while our analysis revealed that the overall number of DAPs between AD and control groups was similar between White and African American individuals, the statistical power, as reflected by the *y*‐axis (‐log10 *p* value), differed between the two groups (Figure 7A and B). This difference in statistical power could be attributed to variations in sample size, biological variability, or other factors between the groups. A small number of changes unique to AD samples of one race were noted (Figure 7E, F), including proteins known to have distinct expression patterns in African American and European American populations.^83^ To further dissect the global changes in protein abundance, we categorized the DAPs into four main subgroups based on their abundance patterns across the racial groups. Some of the proteins represented in each of these groups are illustrated in Figure 7D‐G:

**Group 1**: Proteins exhibiting low abundance in AD irrespective of race. This subgroup includes proteins that show a consistent reduction in abundance in AD samples from both African American and White individuals compared to controls. This group includes proteins like VGF, NRN1, NPTX2, and RPH3A, which have been reported to be neuroprotective against AD.^84^, ^85^, ^86^ The universal downregulation of these proteins suggests a loss of neuroprotective mechanisms in AD, affecting both racial groups similarly.

**Group 2**: Proteins with differential abundance in AD only in the White population. This subgroup includes proteins that are significantly altered in abundance in White individuals with AD but do not show a corresponding change in African American individuals. For instance, proteins associated with immune function (IGHG4) and myoglobin (MB) exhibit high abundance in White AD brains compared to controls. Additionally, proteins such as RNA‐binding protein (RBM3) and ubiquitin (PCMTD2) show low abundance specifically in White AD brains. These findings suggest that certain immune‐related and metabolic processes may be more prominent or exclusively altered in the White population with AD.

**Group 3**: Proteins with differential abundance in AD only in the African American proteome. Similar to Group 2, these proteins are significantly altered in abundance in African American individuals with AD, with no comparable change in the White population. This group includes proteins such as BPHL, which shows low abundance in African American AD brains, and proteins associated with neuroinflammation (CXCL16) and cell signaling (RGN), which have high abundance in this population.^87^, ^88^ These proteins might reflect unique pathological processes or protective factors within the African American community that are not observed in the White population.

**Group 4**: Proteins exhibiting high abundance in AD irrespective of race. This final subgroup includes proteins that are consistently upregulated in AD samples from both African American and White individuals compared to controls. This group includes several hub proteins in the matrisome, such as SMOC1, CTHRC1, SFRP1, and SPON1, which are highly abundant in neuritic plaques and CAA and are colocalized with Aβ.^21^, ^31^, ^55^ The universal increased level of these proteins highlights their central role in AD pathology and suggests they may serve as key biomarkers or therapeutic targets in both racial groups.

In summary, our global differential abundance analysis explored differences in protein levels in the brains of African American and White individuals across AD pathology. The findings showed that most changes in protein abundance were similar across both racial groups. However, some proteins showed race‐specific changes, suggesting that certain biological processes may differ between the two groups. These results highlight both shared and unique aspects of AD across race, offering insights into potential biomarkers and therapeutic targets.

## 4 DISCUSSION

Here we presented a comprehensive large‐scale deep proteome analysis on 1105 DLPFC and 280 STG brain tissues. This dataset consists of approximately 10,000 proteins quantified in a racially and ethnically diverse cohort of AD and controlled aging brain tissues. In addition, QC measures were implemented to ensure the validity of our dataset for subsequent analysis. Consistently with the literature,^21^, ^39^, ^40^, ^43^, ^44^ our analysis identified top proteins associated with sex, race, and AD diagnosis. Additionally, quantified levels of MAPT and APP showed strong associations with neuropathology scores of Braak and CERAD, respectively. Moreover, with minor exceptions, the protein abundance of APOE4 was highly consistent with *APOE* genotyping of the measured samples.

Our analysis demonstrates that the core pathological processes of AD, as reflected by key proteins associated with the disease, are consistent across racial groups. Our proteomic analysis also revealed no significant racial differences in the abundance of Aβ40, a peptide closely associated with cerebral Aβ angiopathy (CAA). This finding is consistent with previous studies that demonstrated similar prevalence and histopathologic characteristics of CAA among African Americans and Caucasians.^89^, ^90^ In addition to Aβ40, Aβ42, which is highly enriched in amyloid plaques, also had no race‐associated differences among African Americans and White individuals. This suggests that the degree of plaque pathology is similar across races, consistent with recent amyloid positron emission tomography (PET) findings showing that African Americans and White individuals have a similar rate of amyloid PET positivity.^91^ Other neuropathological studies of the racially diverse brain tissues have also confirmed a similar pattern of amyloid plaques, morphology, and quantifications in brain tissues of African Americans compared to White individuals.^90^, ^92^ This suggests that the fundamental mechanisms driving core AD pathology, such as the accumulation of Aβ plaques and tau neurofibrillary tangles, are universally shared across diverse populations.

Importantly, in contrast to CSF data published by our group and others, MAPT protein levels in brain tissues (DLPFC and STG) are not significantly lower in African American individuals with AD.^13^, ^14^ Therefore, differences in CSF MAPT levels between African Americans and Whites may not be driven by variations in tau tangle deposition or neurodegeneration in the brain. This suggests that other mechanisms may contribute to the lower peripheral tau levels in African American CSF. In addition, we observed that both African American and White individuals with AD exhibited significant increases in key proteins involved in AD pathology, including proteins associated with the matrisome, neuroinflammation, and synaptic function. These findings are in line with established knowledge of AD pathology,^21^, ^31^, ^55^ underscoring the robustness of these molecular signatures in the brain, regardless of race. The consistency in these core pathologies highlights that the fundamental biological processes underlying AD are preserved across different racial backgrounds.

While the directionality of changes in the proteome was similar between African American and White individuals, with strong overall agreement in the direction of differential abundance, some unique variations were noted, with a small number of proteins exhibiting differential abundance unique to either the White or African American proteome. Proteins such as IGHG4 and MB showed higher abundance, while RBM3 and PCMTD2 exhibited lower abundance in White AD brains, indicating distinct immune and metabolic alterations in this population.^93^, ^94^ In contrast, African American AD brains showed lower levels of BPHL and higher levels of CXCL16 and RGN, highlighting unique neuroinflammatory and cell signaling pathways in this group.^87^, ^88^ These unique changes might be attributable to differences in genetic ancestry, which may influence susceptibility to specific AD‐related pathways. Additionally, comorbidities and factors relating to social determinants of health may also play a role in shaping the proteomic landscape of AD across different racial groups. It is an ongoing priority of the AMP‐AD consortium and other groups to explore the extent to which social determinants of health, such as variations in access to healthcare and education, as well as environmental exposures, may modulate AD risk and progression.

To that end, this study serves as an important data resource for exploring difference in the brain proteome and is complemented by ongoing efforts of the AMP‐AD diversity initiative to share paired genomes and transcriptomes from the same donors.^16^, ^26^ Moving forward, these unique, large datasets derived from diverse populations will set the stage for future investigations aimed at addressing existing knowledge gaps and advancing our understanding of AD pathogenesis across, age, sex, race, and ethnicity. In what follows, we describe several use cases in which this proteomic dataset can be used to address these gaps.

### 4.1 Network analysis

Unbiased proteomics of the human brain in AD, coupled with network analysis, is a valuable approach to organizing and reducing large‐scale, complex protein expression matrix data into groups or “modules” of proteins that highly correlate across tissues.^29^, ^32^, ^95^ We and others have shown that these modules reflect various biological functions with cell‐type specificity linked to AD pathology.^21^, ^22^, ^32^, ^96^ Using this approach modules could reveal potential associations between sex, race, APOE genotype, and AD diagnosis, shedding light on intersecting biological processes that contribute to disease susceptibility. Furthermore, bulk RNA sequencing (RNA‐seq) analysis will be available for the majority of these same tissues profiled by proteomics, which will allow for integrated network analyses to compare transcript expression to protein‐level abundance, which are not generally well correlated in human brain tissues.^21^, ^23^ Ultimately, the development of robust biomarkers and therapeutic targets, generalizable to whole populations, will necessitate integration of multiple data types derived from diverse populations.

### 4.2 Mapping post‐translational modifications

The phosphorylation of tau and proteolytic cleavage of APP into Aβ species are pathological hallmarks of AD and have important roles in disease progression and pathogenesis.^48^, ^49^ Other post‐translational modifications (PTMs) have also been described as altering the brain proteome in AD.^97^, ^98^ Although we did not specifically enrich proteins with antibodies or chemical approaches like immobilized metal affinity chromatography for phosphorylated peptides, the raw MS data can be re‐searched to determine whether high abundance PTMs like phosphorylation, acetylation, methylation, and ubiquitination of tau are altered in these tissues across race. The analysis presented here, therefore, will provide the basis for additional in‐depth interrogation of the AD‐related brain proteome.

### 4.3 Proteogenomics

The AMP‐AD consortium is committed to making the paired whole genomes of the majority of the tissues profiled by proteomics in this study available. Notably, this will offer an opportunity to investigate protein quantitative trait loci (pQTLs) to estimate the effects of genetic variants on protein abundance.^99^ Furthermore, integrating AD genome‐wide association studies (GWASs) with these pQTLs can be used to identify causal genes that confer AD risk through their effects on brain protein abundance. This approach is referred to as proteome‐wide association studies (PWASs), which can now be done with African American GWAS summary statistics on AD or related dementias.^100^, ^101^ Incorporating quantitative measures of genetic ancestry will help to further resolve inherited contributions to AD pathogenesis. Additionally, paired RNA‐seq and proteomics from these same tissues can also be used to identify splicing defects in AD that generate alternative protein isoforms occurring in the brain across different disease states and ancestries.^102^, ^103^ Understanding how alternative splicing contributes to AD pathophysiology and its intersection with demographic factors may uncover novel disease mechanisms and identify splice variants as potential biomarkers or therapeutic targets.

### 4.4 Limitations and future directions

While our study provides valuable insights into the proteomic landscape of AD in a diverse population, several caveats and limitations should be considered. First, it is essential to acknowledge that proteomics data represent a snapshot of protein abundance at end‐stage disease and do not capture dynamic changes in protein expression over the course of disease progression. Additionally, although efforts were made to minimize technical variability through rigorous QC measures, the inherent complexity of brain tissues and potential confounding factors such as comorbidities may introduce biases or artifacts into the dataset. In addition, a few discrepancies were noted; for example, the number of controls in our study was not matched with the number of AD cases, resulting in fewer control cases. Moreover, the lack of *post mortem* interval (PMI) information for all samples is another limitation of our study. Without PMI data, we were unable to account for the potential effects of PMI, even though our previous studies showed a minor impact of PMI on data variance.^21^, ^32^, ^104^ It is also important to note that the interpretation of race‐specific protein changes should be approached with caution, as the biological and genetic basis underlying these differences remains incompletely understood. Further validation studies and replication in independent cohorts are warranted to confirm and extend our findings. Future proteomic studies on the biofluids from the CSF and plasma of diverse participants will be warranted to understand how these changes in the *post mortem* brain are reflected in the periphery and whether they are of prognostic utility. Ultimately, integrated multi‐omic datasets across tissues and biofluids will be needed for further investigation into how AD heterogeneity varies across different ethnoracial backgrounds.

## 5 CONCLUSIONS

In conclusion, this large‐scale deep proteome analysis represents a valuable resource for future exploration of the complexities of AD across diverse ethnoracial groups. While our findings highlight that the core AD‐related pathologies are consistent across populations, the differences observed in certain CSF biomarkers suggest the need for further research. These variations may be influenced by comorbidities, genetic factors, and social determinants of health, underscoring the importance of continued investigation.

## CONFLICT OF INTEREST STATEMENT

The authors declare no conflicts of interest. Author disclosures are available in the supporting information.

## CONSENT STATEMENT

All relevant ethical guidelines have been followed, and any necessary Institutional Review Board and/or ethics committee approvals were obtained. Written informed consent was obtained from all participants before inclusion in the study.

## Supporting information



Supporting Information

Supporting Information

Supporting Information

Supporting Information

Supporting Information

Supporting Information

Supporting Information

Supporting Information

Supporting Information

Supporting Information

## Data Availability

Raw data files and clinical metadata are available at https://doi.org/10.7303/syn53420674. Search results and database, sample‐to‐TMT channel information, and normalized data are available at https://doi.org/10.7303/syn55225561. Summary data are also available at the ShinyApp https://telomere.biochem.emory.edu/diversity/.
