# Supplementary material for: Large‐scale deep proteomic analysis in Alzheimer's disease brain regions across race and ethnicity
Source: Alzheimers Dement. 2024 Nov 13;20(12):8878–97. doi: 10.1002/alz.14360 (PMC11667503; doi:10.1002/alz.14360)
Supplement: Supplementary file 2 — Supporting Information [file ALZ-20-8878-s008.pdf]

fig S1

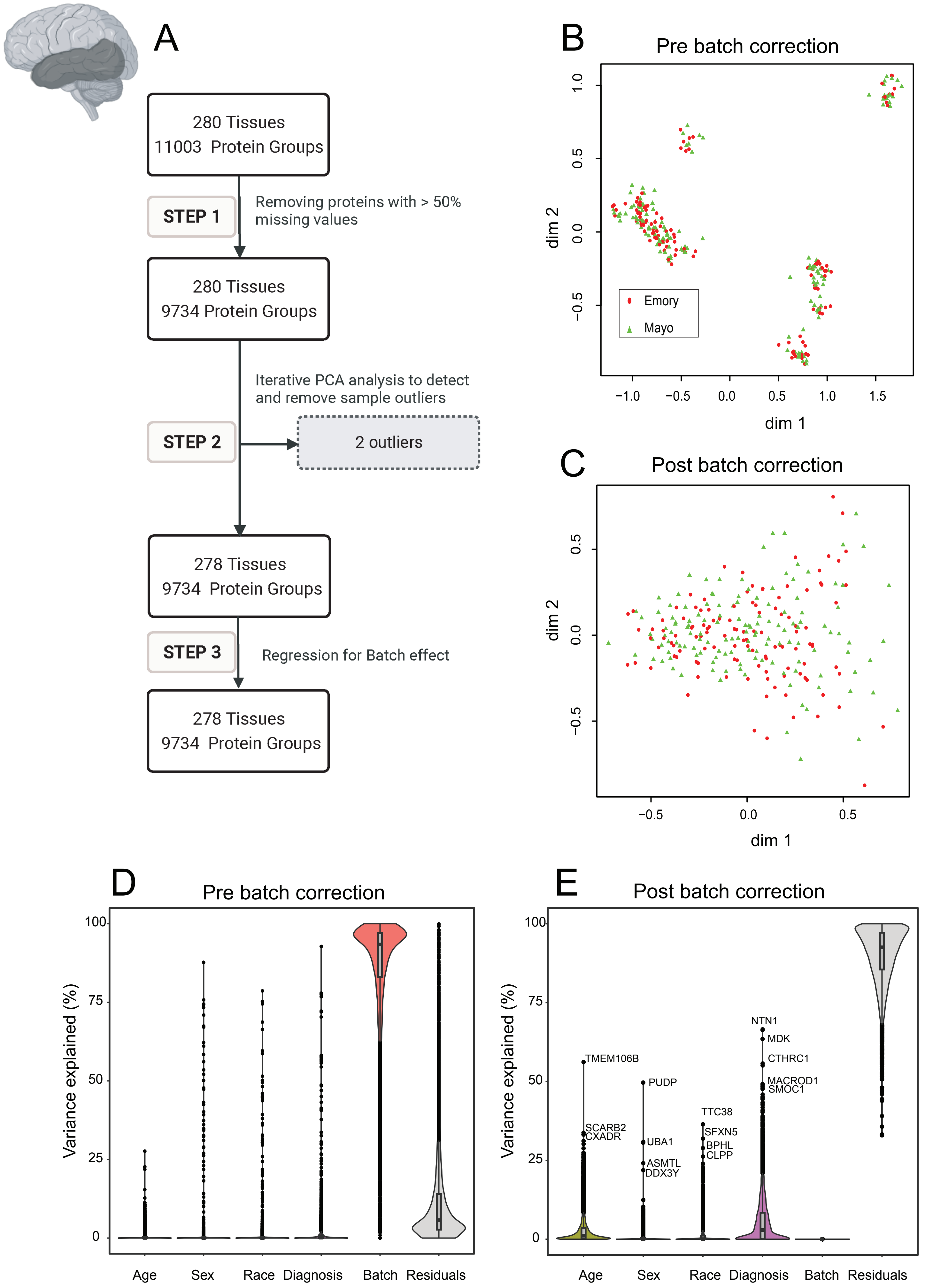

Fig. S1. Quality Control (QC) and Batch Correction for STG Tissue proteins. **A**. The analysis workflow for data QC is depicted in three main steps: Step 1. Handling missing values: Proteins with missing data in more than 50% of the samples were removed, adjusting for sample loading differences through ratio calculation and log2 transformation. This yielded 9,734 proteins across 280 samples. Step 2. Identification and removal of outliers: Iterative principal component analysis (PCA) was utilized to detect and eliminate sample outliers. Following three rounds of PCA, two outliers were removed, resulting in 9,734 proteins across 278 samples. Step 3. Batch effect removal: Regression was applied to mitigate batch effects for the 9,734 proteins in 278 samples. B and C. Analysis of Multidimensional Scaling (MDS) plots: MDS plots depict sample variation (**B**) before batch correction and (**C**) after regression for batch effect. Emory (red) and Mayo (green) samples form distinctive clusters, with some scattering observed among samples before batch regression (**B**). (**C**) demonstrates the impact of batch regression, revealing a more cohesive grouping of Emory (red) and Mayo (green) samples. The correction effectively reduces the dispersion observed in panel B. D and E. Variance partition analysis for proteomic samples: Violin plots (**D**) before and (**E**) after batch correction show the distribution of explained variances in overall proteomic values. Panel D's Y-axis represents the percentage of explained variance, while the X-axis includes factors like age, sex, race, diagnosis, residuals, and batch. Similar to Fig 2.D, batch variance revealed a high impact on the proteomic profile before correction. Panel **E** displays the same factors after batch correction, demonstrating a substantial reduction in variance associated with batch. In addition, after batch correction, age, sex, race, AD diagnosis, and other individual characteristics (residuals) remain influential factors shaping protein abundance patterns. Each data point represents a unique protein, with the corresponding protein names provided adjacent to the top points.
