# Supplementary material for: Large‐scale deep proteomic analysis in Alzheimer's disease brain regions across race and ethnicity
Source: Alzheimers Dement. 2024 Nov 13;20(12):8878–97. doi: 10.1002/alz.14360 (PMC11667503; doi:10.1002/alz.14360)
Supplement: Supplementary file 3 — Supporting Information [file ALZ-20-8878-s010.pdf]

fig S2

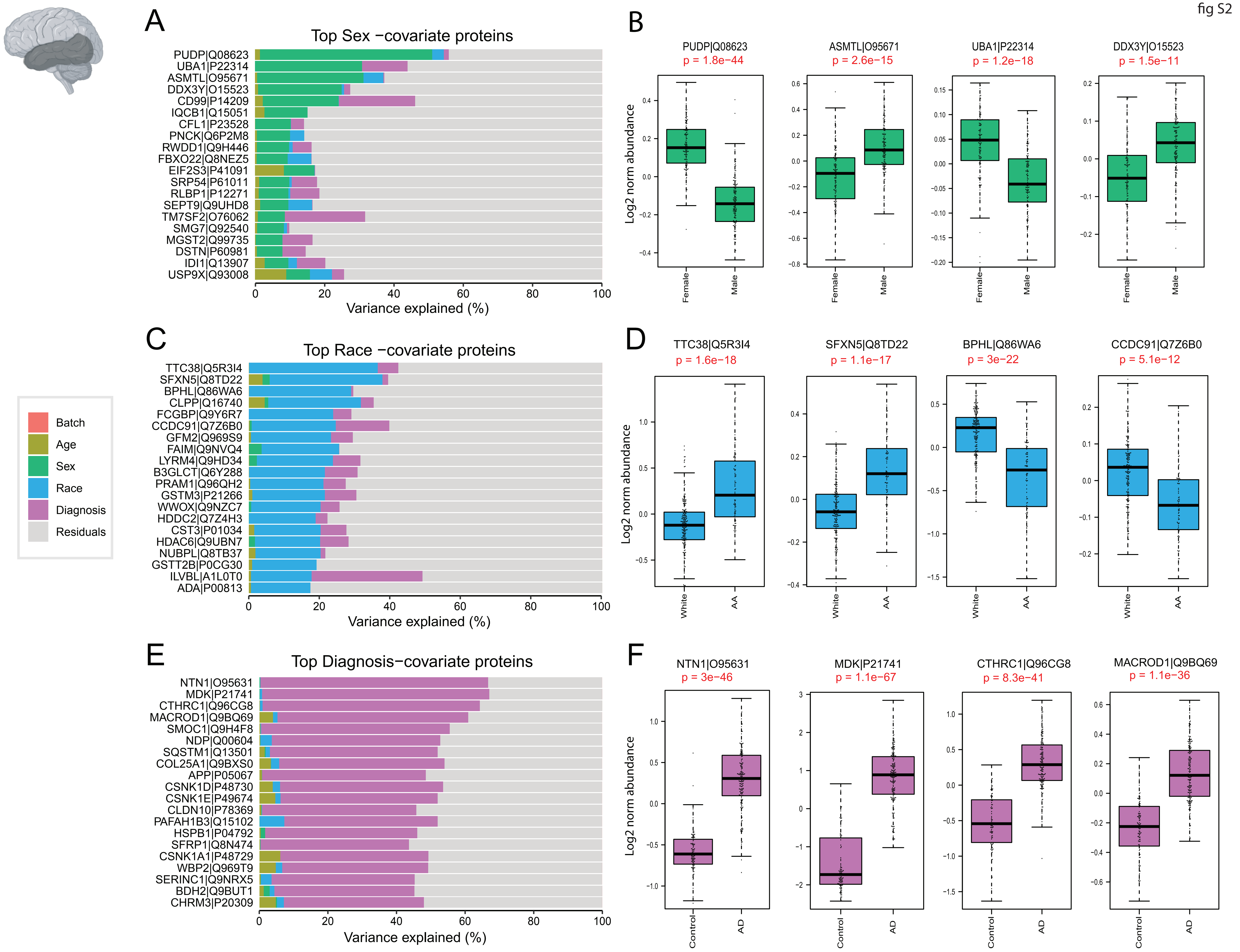

Fig. S2. Variance Explained by individual Characteristics in STG Tissues. The bar plots in panels **A, C**, and **E** illustrate the partitioning of total variance for each protein into fractions attributable to different dimensions of variation in the STG samples. **A**. Top-ranking proteins contributing to sex differences in the dataset were identified through variance partitioning and are presented as bar plots, showing the proportion of variance attributable to sex. Boxplots in panel **B** demonstrate the log2 normal abundance levels of four selected proteins exhibiting significant differences between males and females ($p < 0.05$). **C**. Bar plots display the top proteins with fraction of total variance attributed to race differences in the STG dataset. Boxplots in panel D illustrate the log2 normal abundance levels of four selected proteins demonstrating significant differences between African American individuals and individuals of other races ($p < 0.05$). **E**. Bar plots identify the top AD-associated proteins with fraction of total variance attributed to AD diagnosis within the STG dataset. Boxplots in Panel **F** display the log2 normal abundance levels of four selected proteins exhibiting significant differences between AD samples and controls, as well as other diagnostic categories ($p < 0.05$).
