# Supplementary material for: Large‐scale deep proteomic analysis in Alzheimer's disease brain regions across race and ethnicity
Source: Alzheimers Dement. 2024 Nov 13;20(12):8878–97. doi: 10.1002/alz.14360 (PMC11667503; doi:10.1002/alz.14360)
Supplement: Supplementary file 4 — Supporting Information [file ALZ-20-8878-s003.pdf]

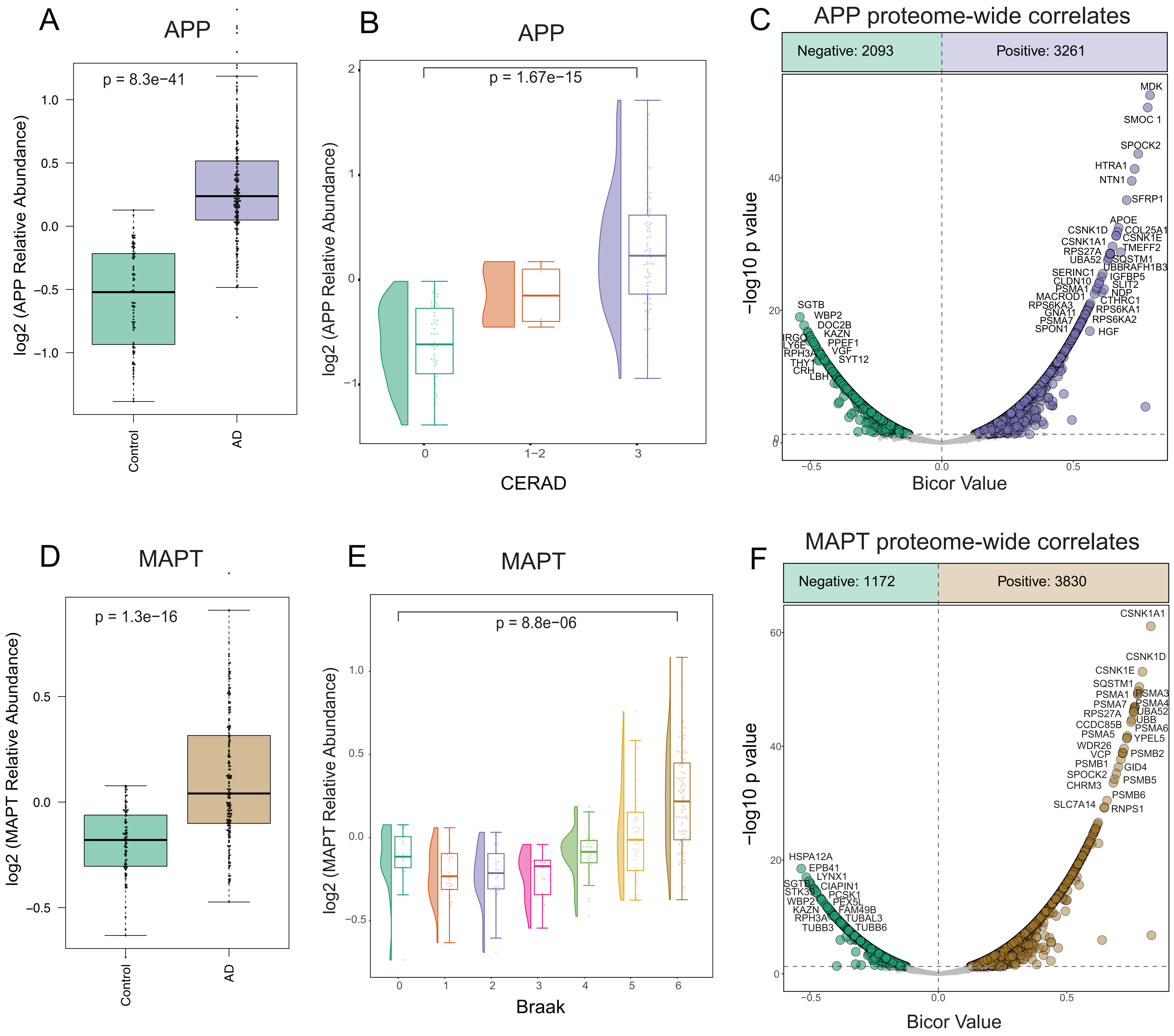

Fig. S3. Correlation of proteomic measurements of Tau and APP with Braak and CERAD pathological scores, as well as with other proteins, in STG samples. (**A**) Box plots show the relative abundance of APP in AD (pink) versus control (green) within STG tissue samples, with statistical significance determined by adjusted ANOVA ($p < 0.05$). (**B**) Raincloud plots depict the distribution of APP levels (Y-axis) across different CERAD stages (X-axis) in the STG region. Given the limited number of samples with CERAD scores of 1 and 2 (n=5), these were combined into a single category. The stages are color-coded as follows: score 1: green, scores 1-2: orange, and score 3: purple. The data indicates a stepwise increase in APP abundance with higher CERAD scores. (**C**) Bicor analysis reveals correlations between APP and 9,734 proteins in the STG. Proteins with significant positive correlations ($p < 0.05$) with APP are shown in purple, those with significant negative correlations ($p < 0.05$) in green, and non-significant correlations in grey. Of the proteins analyzed, 3,261 were positively correlated with APP, while 2,093 were negatively correlated. **D**. Box plots illustrate MAPT levels in STG samples, comparing AD (brown) and control (green) groups, with significance determined by adjusted ANOVA ($p < 0.05$). **E**. Raincloud plots demonstrate the variation in MAPT levels (Y-axis) across Braak stages (X-axis) within STG tissues. The Braak stages are color-coded as follows: score 1: dark green, score 2: orange, score 3: purple, score 4: pink, score 5: light green, score 6: yellow, and score 7: brown. The results show higher MAPT levels in later Braak stages, corresponding to increased tau pathology.
F. Proteome-wide pairwise correlation analyses with Bicor between MAPT and 9,734 proteins in the STG region. Proteins with significant positive correlations ($p < 0.05$) are depicted in brown, while those with significant negative correlations ($p < 0.05$) are shown in green. Grey indicates proteins without significant correlations. Out of the proteins analyzed, 3,830 were positively correlated with MAPT, and 1,172 were negatively correlated.
